# An Integrative Pan-Cancer Analysis Revealing LCN2 as an Oncogenic Immune Protein in Tumor Microenvironment

**DOI:** 10.3389/fonc.2020.605097

**Published:** 2020-12-23

**Authors:** Wen-Xiu Xu, Jian Zhang, Yu-Ting Hua, Su-Jin Yang, Dan-Dan Wang, Jin-Hai Tang

**Affiliations:** ^1^ Department of General Surgery, The First Affiliated Hospital of Nanjing Medical University, Nanjing, China; ^2^ Department of Gastroenterology, Wuxi People’s Hospital Affiliated to Nanjing Medical University, Wuxi, China

**Keywords:** lipocalin 2, pan-cancer, database, immune infiltration, tumor microenvironment

## Abstract

**Background:**

Lipocalin 2 (LCN2), an innate immune protein, plays a pivotal role in promoting sterile inflammation by regulating immune responses. However, the role of LCN2 in diverse cancers remains poorly defined. This research aimed to investigate the correlation between LCN2 expression and immunity and visualize its prognostic landscape in pan-cancer.

**Methods:**

Raw data in regard to LCN2 expression in cancer patients were acquired from TCGA and GTEx databases. Besides, we investigated the genomic alterations, expression pattern, and survival analysis of LCN2 in pan-cancer across numerous databases, including cBioPortal and GEPIA database. The correlation between LCN2 expression and tumor immune infiltration was explored *via* TIMER, and we utilized CIBERSORT and ESTIMATE computational methods to assess the proportion of tumor-infiltrating immune cells (TIICs) and the amount of stromal and immune components from TCGA database. Protein–Protein Interaction analysis was performed in GeneMANIA database, and gene functional enrichment was performed by Gene Set Enrichment Analysis (GSEA).

**Results:**

On balance, tumor tissue had a higher LCN2 expression level compared with that in normal tissue. Elevated expression of LCN2 was related to poor clinical regimen with OS and RFS. There were significant positive correlations between LCN2 expression and TIICs, including CD8+ T cells, CD4+ T cells, B cells, neutrophils, macrophages, and dendritic cells. Moreover, markers of TIICs exhibited different LCN2-related immune infiltration patterns. GSEA analysis showed that the expression of LCN2 was related to retinol metabolism, drug metabolism cytochrome P450 and metabolism of xenobiotics by cytochrome P450.

**Conclusions:**

These findings suggested that LCN2 might serve as a biomarker for immune infiltration and poor prognosis in cancers, shedding new light on therapeutics of cancers.

## Introduction

Lipocalin 2 (LCN2), a novel immune-related gene, belongs to the lipocalin family and has emerged as a pleiotropic modulator during physiological and inflammatory conditions (1). LCN2 has several synonyms including oncogenic lipocalin, neutrophil gelatinase-associated lipocalin (NGAL), uterocalin, 24p3, and siderocalin. The different names are denominated by the tissue where its expression was initially detected or its predicted functions (2). Besides, it can be secreted by adipocytes (3, 4), tumor cells and immune cells (neutrophils and macrophages) (5). Upregulation of LCN2 was observed in a variety of cancers, such as lung cancer, breast cancer, prostate cancer, pancreatic cancer and esophageal cancer (6–9). Currently, LCN2 has received considerable attention as both a promising biomarker and vital mediator of various human cancers, but the relevance of LCN2 function with the tumorigenesis is still unknown.

Cancer is a complicated disease involving interactions between tumor and immune system. Tumor microenvironment (TME) comprises a variety of cells, among which infiltrating immune cells make up a large proportion (10). TME plays a pivotal role in the initiation and development of human cancers. However, it still remained an elusive challenge in comprehending the dynamic regulation mechanism of the stromal and immune components in TME. The tumor–immune cell interaction came into focus as the development of the immunotherapies with immune checkpoint blockade and other strategies, such as therapeutic vaccines and engineered T cells (11),. As an alternative to classic anticancer therapies, immunotherapy has demonstrated efficacy in multiple cancer types and been developed to reactivate adaptive and innate immune systems, which targets interactions between immune cells and tumor cells (12). Currently, a myriad of checkpoint-blocking drugs are applied in cancers, such as anti-CTLA-4, anti-PD-L1, and anti-PD-1 (13). In consequence, there is an urgent need to clarify the immunophenotypes of tumor-immune interactions and validate the new immune-related therapeutic targets in cancers.

In this research, data-mining analysis based on various databases, we comprehensively analyzed the expression of LCN2 and its association with tumor-infiltrating immune cells (TIICs) and related immune markers, and further visualized its prognostic landscape in pan-cancer. This study was designed and performed according to the flow chart (**Figure S1**) The findings implied that LCN2 influenced the prognosis of cancer patients, probably through its interaction with TIICs. LCN2 showed an oncogenic effect on pan-cancer, and elevated LCN2 expression was detrimental to the survival time of human cancer patients. Taking these facts together, LCN2 was not only a marker of immune infiltration and poor prognosis, but also a candidate and promising therapeutic target for cancers.

## Materials and Methods

### Raw Data Acquisition and Processing

TCGA (The Cancer Genome Atlas) research network has profiled and analyzed a large collection of clinical and molecular data of over 10,000 tumor patients across 33 different tumor types (14, 15). Transcriptome RNA-seq data of 33 cancers were extracted from TCGA database (https://portal.gdc.cancer.gov/). 33 cancer types were included: ACC, BLCA, BRCA, COAD, DLBC, ESCA, GBM, HNSC, KICH, KIRC, KIRP, LAML, LGG, LIHC, LUAD, LUSC, OV, PAAD, PRAD, READ, SKCM, STAD, TGCT, THCA, THYM, UCEC, and UCS.

### Genomic Alterations of LCN2 in Cancers

Alteration of LCN2 status in cancer patients was acquired from the online cBioPortal database (http://www.cbioportal.org/) for cancer genomics (16). The genomic alterations of LCN2 included copy number amplification, deep deletion, missense mutation with uncharted significance and mRNA upregulation.

### Analysis of LCN2 Expression in Cancers

The information of differential expression of LCN2 between tumor and matched normal tissue was from TCGA and Genotype Tissue Expression (GTEx) projects. GTEx (http://gtexportal.org) is a tissue bank and data resource set up by the National Institutes of Health (NIH) Common Fund, and 53 human normal tissues in the aggregate from approximately 1,000 individuals have been studied by genetic variation, RNA sequencing, and other molecular phenotypes (17). Regarding parameter selection, we chose log2 (TPM+1) transformed expression data for plotting.

### Survival Analysis and Relationship With Clinical Stage

Gene Expression Profiling Interactive Analysis (GEPIA) (http://gepia.cancer-pku.cn) (18) database is an online platform for dissecting the RNA sequencing expression data from the TCGA and the GTEx projects, using a standard processing approach. “Survival” module of GEPIA was utilized to assess the correlation between LCN2 expression and prognosis of cancers. GEPIA also provided interactive functions such as profiling according to pathological stages (18).

### Relationship Between LCN2 Expression and Immunity

TIMER (Tumor Immune Estimation Resource) database (https://cistrome.shinyapps.io/timer/) (19) is an integrated web server to evaluate of the abundance of TIICs across diverse cancer types. We next explored the relationship between the level of LCN2 expression and the abundance of TIICs, including CD4+ T cells, CD8+ T cells, B cells, neutrophils, dendritic cells (DCs), and macrophages. Moreover, the database can also accurately quantify the purity of tumors. In addition, we explored the differences of immune cell subtypes. Cell-type identification by Estimating Relative Subsets of RNA Transcripts (CIBERSORT) algorithm was applied to assess relationship between LCN2 expression and 22 immune cell subtypes based on expression file. Gene markers of TIICs were analyzed including the markers of T cells (general),CD8+ T cells, B cells, monocytes, TAMs, M1 macrophages, M2 macrophages, DCs, neutrophils, natural killer (NK) cells, follicular helper T (Tfh) cells, T-helper 1 (Th1) cells, T-helper 2 (Th2) cells, T-helper 17 (Th17) cells, exhausted T cells, Tregs, and Mast cells (20). These gene markers include BLTA, CD200, TNFRSF14, NRP1, LAIR1, TNFSF4, CD244, LAG3, ICOS, CD40LG, CTLA4, CD48, CD28, CD200R1, HAVCR2, ADORA2A, CD276, KIR3DL1, CD80, PDCD1, LGALS9, CD160, TNFSF14, IDO2, ICOSLG, TMIGD2, VTCN1, IDO1, PDCD1LG2, HHLA2, TNFSF18, BTNL2, CD70, TNFSF9, TNFRSF8, CD27, TNFRSF25, VSIR, TNFRSF4, CD40, TNFRSF18, TNFSF15, TIGIT, CD274, CD86, CD44, and TNFRSF9.

Tumor mutation burden (TMB) is emerging as a new and profound biomarker for predicting immunotherapy effect and is calculated as total amount of mutations per DNA megabases, in which the detected variants are defined as insertions, base substitutions, or deletions across bases (21). Microsatellite instability (MSI), a molecular tumor phenotype, referred to the spontaneous loss or gain of nucleotides from short tandem repeat DNA tracts (22). Analysis regarding relationship between TMB and MSI was conducted by Sangerbox (http://www.sangerbox.com/tool).

Accumulating evidence suggested that tumor immune microenvironment played an important role in development of cancers. In order to set up the association of the estimated proportion of immune and stromal with LCN2 expression, we used Sangerbox online platform to estimate the ratio of immune-stromal component in TME. In addition, results were exhibited in the form of these three kinds of scores: ImmuneScore, StromalScore, and ESTIMATEScore. The higher score estimated in ImmuneScore or StromalScore positively correlated with the ratio of immune or stromal, and it referred to the higher the respective score and the larger the ratio of the corresponding component in TME. ESTIMATEScore was the sum of both, denoting the integrated proportion of both components in TME.

### Protein–Protein Interaction Network Construction

GeneMANIA (http://www.genemania.org) is an interactive and intuitive website for constructing protein-protein interaction (PPI) network, which generates hypotheses about gene function prediction and detects genes with similar functions (23, 24). This network integration algorithm features the following bioinformatics methods: physical interaction, coexpression, colocalization, gene enrichment analysis, genetic interaction and website prediction. In this study, GeneMANIA was applied for PPI analysis of LCN2.

### Gene Set Enrichment Analysis

In order to explore the biological signaling pathway, Gene set enrichment analysis (GSEA) was performed in the high-expression and the low-expression groups compared with the median level of LCN2 expression respectively. The top five terms of KEGG and HALLMARK analyses were exhibited. KEGG pathways with significant enrichment results were demonstrated on the basis of NES (Net enrichment score), gene ratio, and P value. Gene sets with |NES|>1, NOM p <0.05, and FDR q <0.25 were considered to be enrichment significant (25).

### Statistical Analysis

Gene expression data from the TCGA and GTEx databases were analyzed using Student’s t-test. The correlation analysis was evaluated in the TIMER database using Spearman’s correlation analysis. The correlations between LCN2 expression and abundance scores of immune cells evaluated by Spearman’s correlation. All analyses were performed with the R software (version 3.5.1, www.r-project.org) loaded with R package (ggplot2, circlize, clusterProfiler, DOSE and enrichplot) to visualize the results. Results with P <0.05 were considered as statistically significant, providing credibility for the data analysis.

## Results

### The mRNA Expression and Genetic Alteration Differences of LCN2 in Cancers

**Figure 1:**
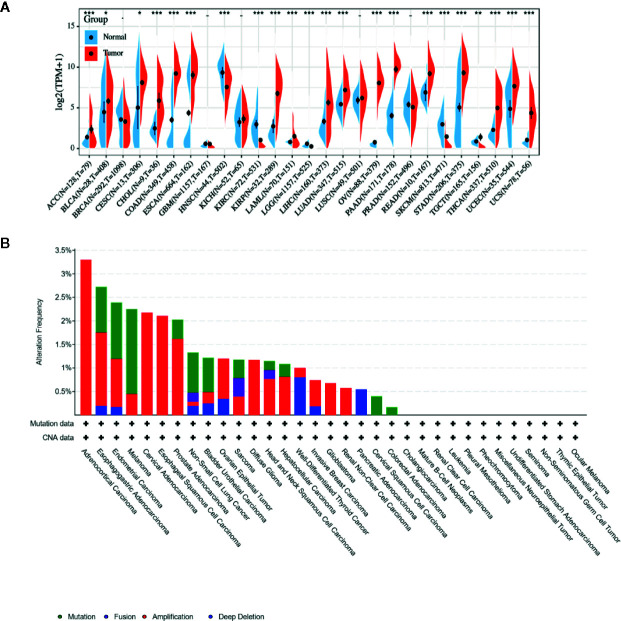
**(A)** Expression level of LCN2 in different cancer types from TCGA and GTEx data. It is clear that there is significant upregulation of LCN2 in ACC, BLCA, CESC, CHOL, COAD, ESCA, KIRP, LIHC, LUAD, PAAD, READ, STAD, THCA, UCEC, and UCS. The red fusiformis represents tumor tissue, and the blue fusiformis represents normal tissue. T, tumor; N, normal; n, number. X axis, number of tumor and normal samples. Y axis, transcript per million [log2(TPM + 1)]. *p < 0.05, **p < 0.01, and ***p < 0.001. **(B)** The genetic alteration type and frequency of LCN2 in various cancers. The cBioPortal database was applied to study the LCN2 mutation in cancers. The results are displayed as a histogram of the alteration frequencies of LCN2 across cancer studies. Color images are available online.

Due to the fact that LCN2 has a potential role as a sensitive indicator in saliva, it might represent an important new target or biomarker for cancer diagnosis. To figure out whether LCN2 expression correlates with cancer, we evaluated LCN2 expression in different tumors and adjacent normal tissues. Data from the TCGA and GTEx database showed that LCN2 mRNA expression was significantly higher in ACC, BLCA, CESC, CHOL, COAD, ESCA, KIRP, LIHC, LUAD, PAAD, READ, STAD, THCA, UCEC, and UCS tumor tissues compared to that in normal tissues, indicating that it might function as an oncogenic molecule in the development of diverse tumors (**Figure 1A**). It has been widely acknowledged that genomic mutation is closely associated with tumorigenesis. To figure out genomic mutation of LCN2 in cancers, comparative analysis of LCN2 was performed. We firstly checked the genetic alterations of LCN2 genes in cancer patients using cBioPortal database. The genetic alteration profiling of LCN2 showed that its amplification was one of the most important single factors for alteration in ACC, cervical adenocarcinoma, ESCC, PAAD, ovarian epithelial tumor, diffuse glioma, HNSC, HCC, BRCA, glioblastoma, renal non-clear cell carcinoma and PAAD. In addition, LCN2 mutation frequencies are the highest in UCEC, Melanoma, NSCLC, BLCA, COAD, and CHOL (**Figure 1B**).

### Prognostic Value of LCN2 in Cancers

**Figure 2:**
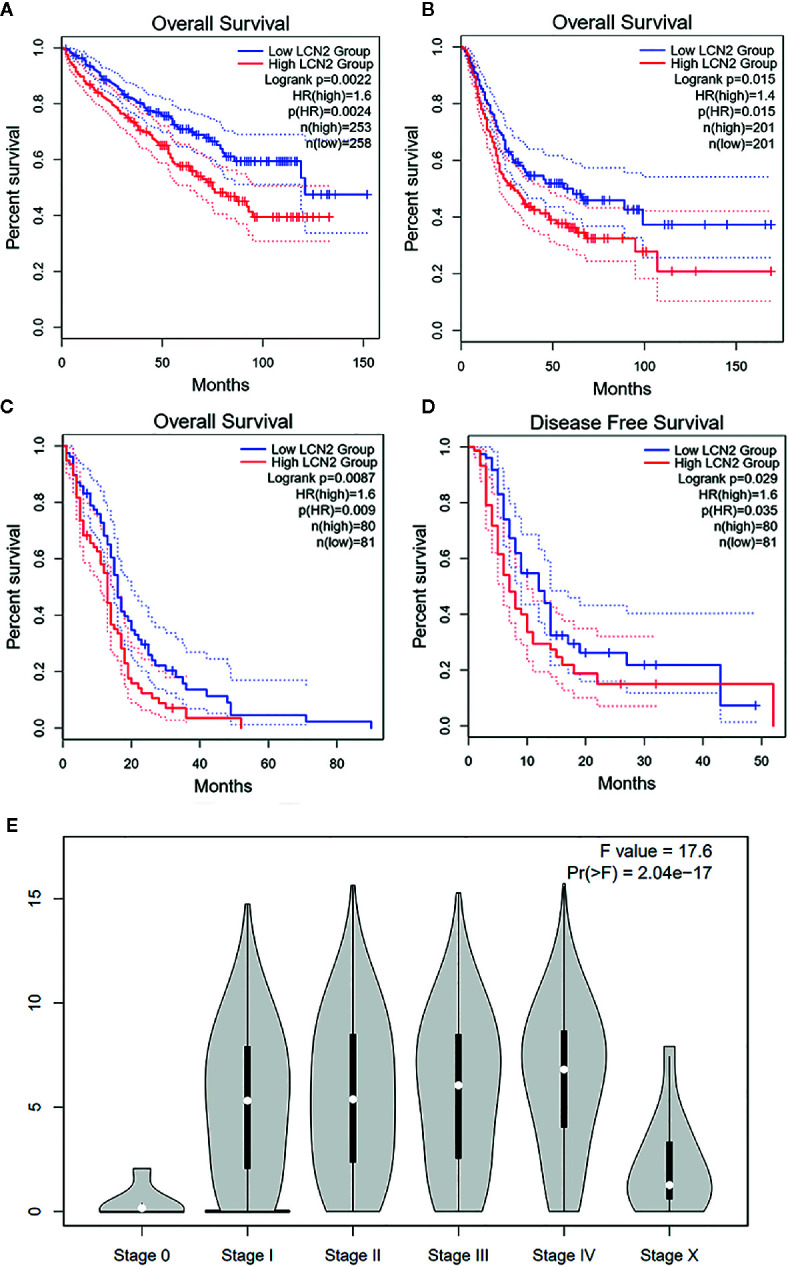
Kaplan–Meier survival curves comparing high and low expression of LCN2 in different cancer types in GEPIA. **(A)** OS in BLCA **(B)** OS in KIRC **(C)** OS in GBM **(D)** DFS in GBM. **(E)** LCN2 expression was positively correlated with advanced stages of cancers (based on GEPIA database). OS, overall survival; DFS, disease-free survival.

Next, we further assessed the prognostic value of LCN2 for pan-cancer (OS and RFS) in GEPIA. Elevated expression of LCN2 is significantly correlated with poor OS and RFS. Particularly, compared with a low expression level, a high expression level of LCN2 was correlated with a worse OS in BLCA (HR = 1.6, P = 0.0024, **Figure 2A**), KIRC (HR = 1.4, P = 0.015, **Figure 2B**) and GBM (HR = 1.6, P = 0.009, **Figure 2C**), and DFS in GBM (HR = 1.6, P = 0.0035, **Figure 2D**). The above data suggested that LCN2 expression level was a great factor affecting the survival of cancers, though their relationship may vary depending on tumor type. In addition, based on the GEPIA dataset, we verified that LCN2 expression had a forceful positive association with advanced cancer stages (P < 0.001, **Figure 2E**).

### Correlation Between LCN2 Expression and Immune Infiltrating Level in Cancers

**Figure 3:**
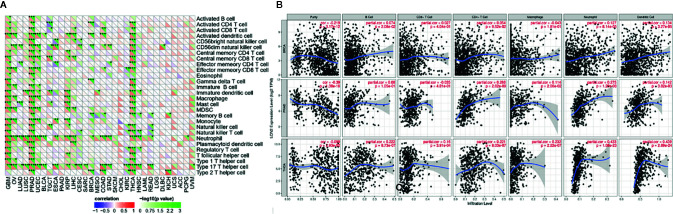
Correlation of LCN2 expression with immune infiltration level in cancers. **(A)** Correlation of LCN2 expression with infiltration level of immune cells by xCell in TCGA. Immune cells positively correlating with LCN2 expression in TCGA dataset were labeled in red, and immune cells negatively correlating with LCN2 expression in TCGA dataset were labeled in violet. **(B)** Correlation of LCN2 expression with immune infiltration level in BRCA, PRAD, and THCA. LCN2 expression has significant negative correlation with tumor purity, and significant positive correlation with infiltrating levels of B cell, CD8+ T cell, CD4+ T cell, macrophage, neutrophil, and dendritic cell. No relation with infiltrating levels of B cell and CD8+ T cell. *p < 0.05, **p < 0.01, and ***p < 0.001.

**Figure 4:**
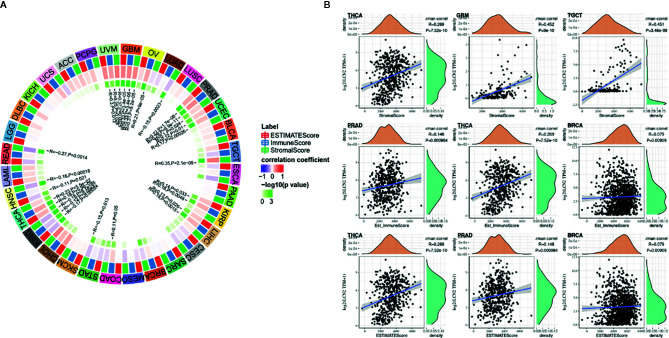
Correlation of scores with LCN2 expression in cancers **(A)** Correlation of the estimated proportion of immune and stromal with LCN2 expression in cancers, and analysis was used by ImmuneScore, StromalScore, and ESTIMATEScore. **(B)** Top three cancers by ImmuneScore, StromalScore, and ESTIMATEScore, respectively. Correlation of ImmuneScore and StromalScore.

TIICs are a significant part of the complex microenvironment that regulate development and progression of diverse cancers (26). The quantity and activity status of tumor infiltrating lymphocytes are important predictive criterion for cancer survival times (27). Hence, we explored the correlation between immune infiltration and LCN2 expression. We determined whether LCN2 expression was associated with the immune infiltration level in various cancers by exploring the coefficient of LCN2 expression and immune infiltration level based on TIMER database. The results indicated that LCN2 expression had significant correlations with tumor purity in 14 cancer types. In addition, LCN2 expression was notably correlated with the infiltration levels of CD4+T cells in 13 cancer types, B cells in 12 cancer types, CD8+T cells in seven cancer types, macrophages in 10 cancer types, neutrophils in 12 cancer types, and dendritic cells in 20 cancer types. The results also revealed that BRCA, PRAD and THCA were three cancer types most strongly correlated with LCN2 expression in immune infiltrating level. In BRCA, the level of LCN2 expression negatively correlated with tumor purity (r = −0.219, P = 3.17e-12), and positively correlated with B cells (r = 0.074, P = 2.08e-02), neutrophils (r = −0.127, P = 8.14e-05), and dendritic cells (r = 0.134, P = 3.27e-05). In PRAD, LCN2 expression negatively correlated with tumor purity(r = −0.39, P = 1.38e-16) and positively correlated with CD4+ T cells (r = 0.288, P = 2.62e-09), macrophages (r = 0.114, P = 2.00e-02), neutrophils (r = 0.275, P = 1.20e-08), and dendritic cells (r = 0.142, P = 3.82e-03). In THCA, LCN2 expression negatively correlated with tumor purity(r = −0.099, P =2. 93e-02), and positively correlated with B cells (r = 0.222, P = 8.73e-07) CD4+ T cells (r = 0.221, P = 8.03e-07), CD8+ T cells (r = 0.16, P = 3.91e-04), macrophages (r = 0.232, P = 2.22e-07), neutrophils (r = 0.433, P = 1.06e-23), and dendritic cells (r = 0.439, P = 2.89e-24) (**Figure 3B**). Further analysis using Sangerbox online tool also showed the correlation between infiltration of 28 kinds of immune cell subtypes and LCN2 expression (**Figure 3A**). Neutrophils and Type 17 T helper cell were two immune cell types most strongly correlated with LCN2 expression across 32 cancer types. Moreover, LCN2 expression in TGCT related positively with activated CD4+ T cell and activated CD8+ T cell infiltration. In addition, UVM, GBM, and PRAD were positively correlated with ImmuneScore, StromalScore, and ESTIMATEScore. On the contrary, KIRC was negatively correlated with these three scores. UCEC, KIRP, LIHC, and THCA were positively correlated with ESTIMATEScore and ImmuneScore. HNSC was negatively correlated with ESTIMATEScore and StromalScore (**Figure 4A**). The top three tumors most significantly correlated with expression of LCN2 were THCA, GBM and TGCT (StromalScore), PRAD, THCA and BRCA (ImmuneScore), THCA, PRAD and BRCA (ESTIMATEScore) respectively (**Figure 4B**). Therefore, the results indicated that LCN2 expression was tightly correlated with the extent of immune infiltration in cancers. Further information was available in the **Supplementary Files 2** and **4**.

### Correlations Between LCN2 Expression and Immune Marker Sets, TMB, and MSI in Cancers

**Figure 5:**
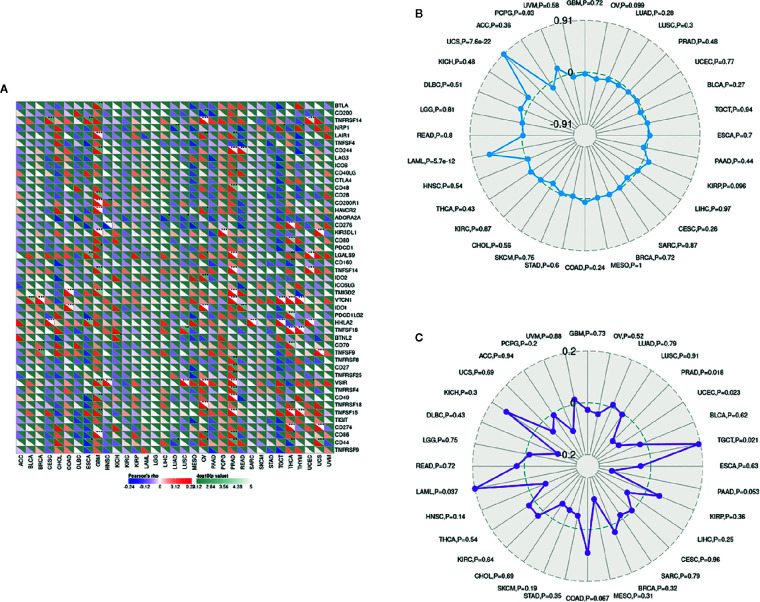
Correlations between LCN2 expression and immunity, including immune marker sets, TMB and MSI in cancers. **(A)** Correlation between LCN2 expression and immune marker sets. **(B)** Radar map of correlation between LCN2 expression and TMB. **(C)** Radar map of correlation between LCN2 expression and MSI.

The importance of immunosurveillance in determining the prognosis of various types of cancers is widely accepted. Tumors could evade immune responses *via* taking advantage of immune checkpoint genes, including PD-1 and CTLA-4. To further examine the association between LCN2 and the extent of immune infiltration in different subtypes of breast cancer, we analyzed the correlation between LCN2 and immune checkpoint gene expression. In PRAD, LCN2 expression was positively correlated with expression of CD244, CD48, LGALS9, TNFSF14, TMIGD2, VTCN1, TNFSF9, TNFRSF8, CD27, TNFRSF25, VSIR, TNFRSF4, CD40, TNFRSF15, CD86, and CD44 (**Figure 5A**). These results suggested that high expression of LCN2 potentially played a vital role in mediating immune evasion. In addition, LCN2 was positively correlated with TMB in BRCA, ESCA, LGG, THCA, and negatively correlated with TMB in OV, PRAD, and SKCM (**Figure 5B**). LCN2 was positively correlated with MSI in KIRC, SARC and TGCT, and negatively correlated with MSI in HNSC, PRAD, and SKCM (**Figure 5C**). Association between LCN2 expression and TMB varied markedly among cancer types. Higher expression of LCN2 was correlated with higher TMB in BRCA, ESCA, LGG, and THCA. Higher somatic TMB was correlated with better OS and an optimal subgroup for ICI therapy in cancer patients (28, 29). All these data together indicated that high LCN2 expression was widely associated with immunity in cancers. The correlations were explored in more detail in **Supplementary Files 3**, **5** and **6**.

### PPI Network of LCN2 in Cancers and Enrichment Analysis

**Figure 6:**
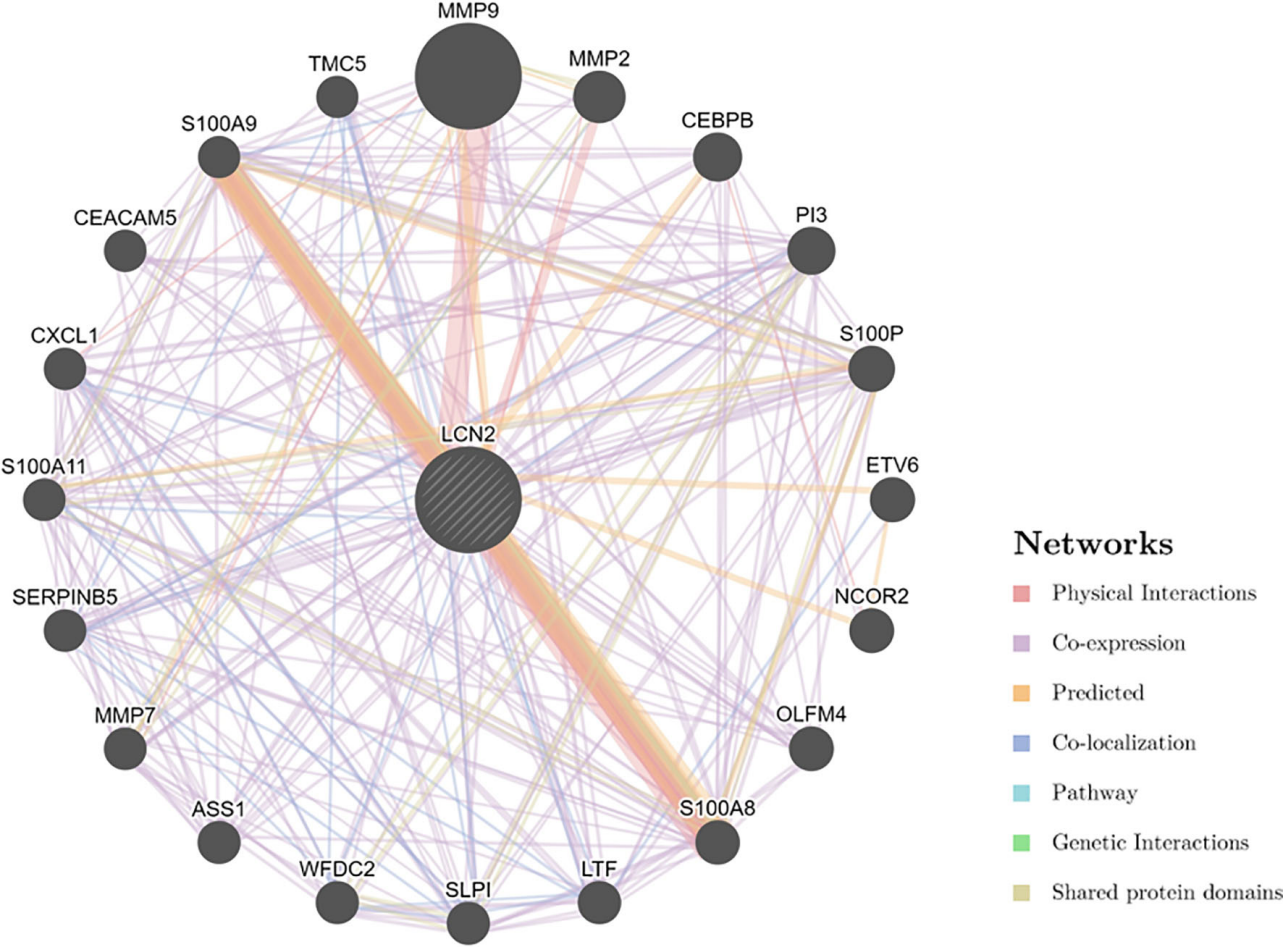
PPI network for LCN2 was constructed in GeneMANIA. Different colors of the network edge indicate the bioinformatics methods applied: physical interaction, coexpression, predicted, colocalization, pathway, genetic interaction, and shared protein domains. PPI, protein–protein interaction.

**Figure 7:**
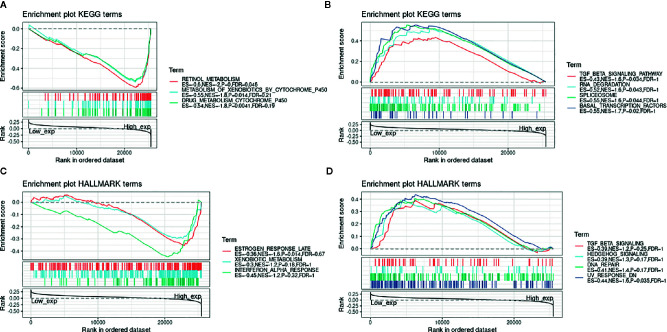
GSEA for samples with high LCN2 expression and low expression. **(A)** The enriched gene sets in KEGG collection by the high LCN2 expression sample. **(B)** The enriched gene sets in KEGG by samples with low LCN2 expression. **(C)** Enriched gene sets in HALLMARK collection, the immunologic gene sets, by samples of high LCN2 expression. **(D)** Enriched gene sets in HALLMARK by the low LCN2 expression. Each line representing one particular gene set with unique color, and up-regulated genes located in the left approaching the origin of the coordinates, by contrast the down-regulated lay on the right of x-axis. Only gene sets with NOM p < 0.05 and FDR q < 0.06 were considered significant. And only several leading gene sets were displayed in the plot.

Next, to explore the potential mechanisms that LCN2 participated in the carcinogenesis of cancers, we used GeneMANIA online tool to construct a PPI network for LCN2, and the result is shown in **Figure 6**. As vividly shown in the picture, LCN2 had strong physical interactions with MMP-9, which is crucial in cancer metastasis. LCN2 constitutes a complex with matrix metalloproteinase-9 (MMP-9), thus increasing its stability and protecting this enzyme from degradation (30). This happens to be consistent with the results of the coexpression. Furthermore, LCN2 was predicted to have significant association with S100A9 and S100A8. Then, GSEA was performed to identify the functional enrichment of high LCN2 expression and low LCN2 expression (**Figure 7**). KEGG enrichment term exhibited that high expression of LCN2 was mainly associated with metabolic-related activities, including metabolism of xenobiotics by cytochrome P450, retinol metabolism and drug metabolism cytochrome P450. However, there was no significant enrichment in HALLMARK terms.

## Discussion

The biological function of LCN2 was proved to be involved in innate immune responses and inflammation tumor microenvironment and promoted malignant development in a wide variety of cancer types (31–33). In addition, the differential expression of LCN2 was especially higher in a series of human epithelial cancers, such as pancreatic, breast, ovarian, thyroid, and colon (9). In pancreatic ductal adenocarcinoma (PDAC), depletion of LCN2 could diminish extracellular matrix deposition, immune cell infiltration, and tumor growth (31). LCN2 was regarded as a vital regulator of tumorigenesis, invasiveness, and metastasis in breast cancer (32, 34). LCN2 secretion by neutrophils and CXCL1-LCN2 paracrine axis conferred malignant phenotypes to prostate cancer cells *via* the Src activation and epithelial-mesenchymal transition (EMT) (35).

Enrichment analysis showed high expression of LCN2 was mainly associated with metabolic-related activities. Metabolic inflammation is distinguished by the dysregulation of cytokine and adipocytokine expression in adipose tissue. Notably, LCN2 can be secreted by adipocytes. LCN2 is an adipokine increased in the visceral adipose tissue and serum of obese individuals (36). Obesity is relevant to increased macrophage infiltration of adipose tissue (37). LCN2 could protect MMP-9 from degradation as previously mentioned (38), and the more active pool of MMP-9 was available to promote angiogenesis by remodeling extracellular matrix (39). In this case, EMT is primarily induced *via* MMP-9-independent pathways.

Under normal circumstances, the immune system can recognize and eliminate tumor cells in tumor microenvironment. However, tumor cells can adopt different strategies to survive and grow, making the immune system restrained. Tumor immunotherapy can restore the body’s normal antitumor immune response, including monoclonal antibody class immune checkpoint inhibitors, cancer vaccines, therapeutic antibodies and cell therapy. TIICs have a clinical impact on patient’s outcome in diverse cancers (13). Here we collected more than 40 common immune checkpoint genes, analyzed the expression relationship between our gene expression and immune checkpoint genes, extracted these immune checkpoint genes respectively, and calculated the correlation with the expression of our target genes. Elevated expression of PD-1 and PD-L1 by TIICs was correlated with poor prognosis and histological grade in cancer patients (40). LCN2 was positively correlated with tumor purity and negatively correlated with TIICs. Up-regulation of LCN2 was correlated with unfavorable prognosis in BLCA, KIRC and GBM. The results revealed that the expression of LCN2 was correlated with the infiltration levels of cancers. MSI was correlated with higher risk of cancer with distinct clinicopathological characteristics, including increased TMB and higher numbers of tumor-infiltrating lymphocytes (41). TMB was a latent biomarker to predict the response to immune checkpoint blockade (29, 42). Additionally, Thomas et al. reported that TMB determined immune-related survival results of breast cancer patients (43). Therefore, our research shed light on understanding the latent role of LCN2 in tumor immunology and its use as a prognostic biomarker of cancers.

However, even though we investigated and integrated information from different databases, there were still some limitations in the current study. To begin with, although the bioinformatic analysis provided us some meaningful insights of LCN2 in cancers, biological experiments *in vitro* or *in vivo* are needed to verify our findings and promote clinical utility. Further mechanistic studies will be beneficial for elucidating the role of LCN2 at the molecular and cellular levels. Secondly, posttranslational modifications are of great significance in regulating intracellular signaling and the activity of regulatory factors (44, 45), but post-translational modification information of LCN2 is not available in these databases. Furthermore, despite the finding that LCN2 expression was correlated with immunity and clinical survival in human cancers, we were not sure that LCN2 influenced clinical survival *via* immune pathway.

In summary, the data in this study elucidated the close correlation and the prognostic significance of LCN2 expression in diverse human cancers. LCN2 might be considered as a novel target for cancer therapy since they showed upregulation in multiple cancers and correlated with worse prognosis. In addition, our results provided insights in the significant role of LCN2 in tumorigenesis and metastasis, providing a potential mechanism that LCN2 expression might modulate tumor immunity, metabolic activity and EMT in cancers. Future prospective studies focusing on LCN2 expression and tumor immune microenvironment could be helpful in giving a definitive answer, thus providing an immuno-based anti-cancer strategy.

## Data Availability Statement

Publicly available datasets were analyzed in this study. This data can be found here: The Cancer Genome Atlas (https://portal.gdc.cancer.gov/).

## Author Contributions

W-XX and JZ conceived and designed this study. W-XX, JZ and Y-TH obtained the data. W-XX, Y-TH, S-JY and D-DW analyzed the data. W-XX, JZ, S-JY, D-DW and J-HT helped discuss the results. W-XX drafted the manuscript. All authors contributed to the article and approved the submitted version.

## Funding

This research was supported by the National Key Research and Development Program of China (No. 2016YFC0905900), National Natural Science Foundation of China (No. 81872365) and Jiangsu Provincial Key Research Development Program (No. BE2019731).

## Conflict of Interest

The authors declare that the research was conducted in the absence of any commercial or financial relationships that could be construed as a potential conflict of interest.

## Supplementary Material

Supplementary File 1: Figure 1 Flow diagram of this study. TIICs, tumor-infiltrating immune cells; PPI, protein-protein interaction; TMB, Tumor mutation burden; MSI, Microsatellite instability.

Supplementary File 2: Text 1 Correlation between LCN2 expression and TIICs.

Supplementary File 3: Text 2 Correlation between LCN2 expression and gene markers of immune cells.

Supplementary File 4: Text 3 Correlation between LCN2 expression and ImmuneScore, StromalScore, and ESTIMATEScore.

Supplementary File 5: Text 4 Correlation between LCN2 expression and TMB.

Supplementary File 6: Text 5 Correlation between LCN2 expression and MSI.
