# Supplementary figures and images for: An Integrative Pan-Cancer Analysis Revealing LCN2 as an Oncogenic Immune Protein in Tumor Microenvironment
Source: Front Oncol. 2020 Dec 23;10:605097. doi: 10.3389/fonc.2020.605097 (PMC7786136; doi:10.3389/fonc.2020.605097)

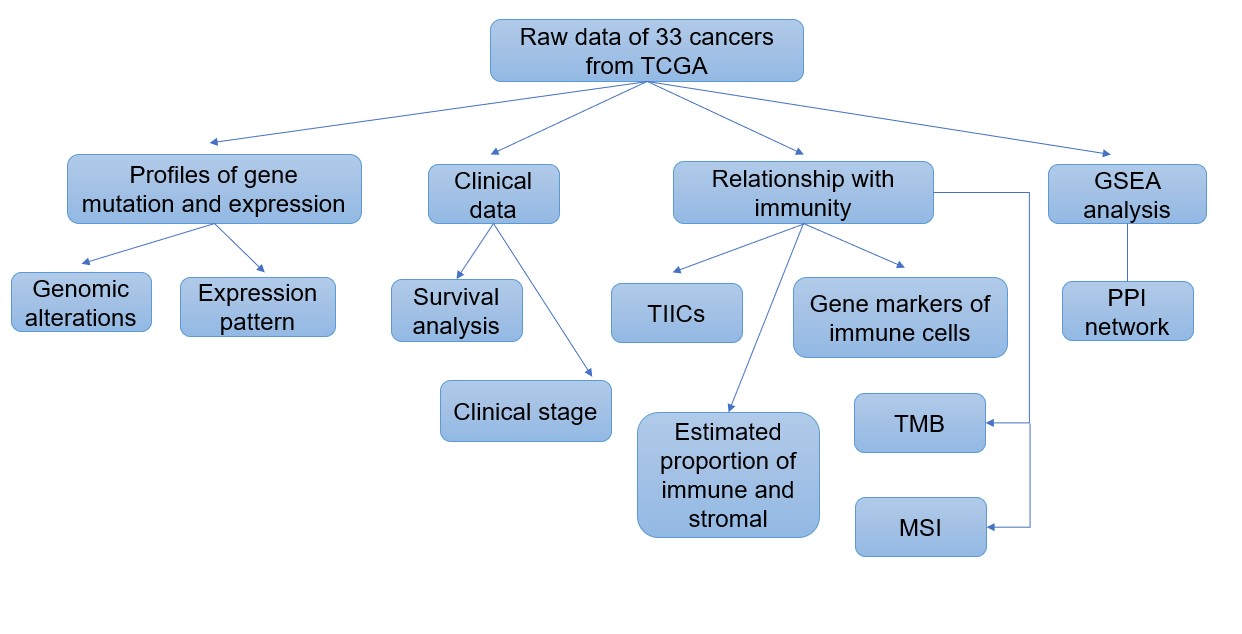

Supplement: Supplementary File 1: Figure 1 — Flow diagram of this study. TIICs, tumor-infiltrating immune cells; PPI, protein-protein interaction; TMB, Tumor mutation burden; MSI, Microsatellite instability. [file Image_1.jpg]
